# Psychosocial correlates of regular syphilis and HIV screening practices among female sex workers in Uganda: a cross-sectional survey

**DOI:** 10.1186/s12981-019-0244-0

**Published:** 2019-09-18

**Authors:** Richard Muhindo, Barbara Castelnuovo, Andrew Mujugira, Rosalind Parkes-Ratanshi, Nelson K. Sewankambo, Juliet Kiguli, Nazarius Mbona Tumwesigye, Edith Nakku-Joloba

**Affiliations:** 1 Department of Nursing, College of Health Sciences, Makerere University, Kampala, Uganda; 2 Infectious Diseases Institute, College of Health Sciences, Makerere University, Kampala, Uganda; 3 Cambridge Institute of Public Health, University of Cambridge, Cambridge, UK; 4 School of Medicine, College of Health Sciences, Makerere University, Kampala, Uganda; 5 School of Public Health, College of Health Sciences, Makerere University, Kampala, Uganda

**Keywords:** Correlates, Regular syphilis and HIV screening, Female sex workers, Uganda

## Abstract

**Background:**

Limited data are available regarding correlates of regular sexually transmitted infections (STIs) and HIV screening among female sex workers (FSW) in Sub-Saharan Africa. In this study, we aimed to assess the frequency of regular syphilis and HIV screening and the psychosocial correlates associated with screening among FSW in Uganda.

**Methods:**

This cross-sectional correlational study was conducted among 441 FSW, aged 17–49 years. We enrolled FSW through peer referrals and ascertained self-reported data on number of serological tests for HIV, syphilis and other STIs in the prior 12 months using an interviewer-administered questionnaire. In addition, we assessed attitudes, norms, social influences and self-efficacy towards 3-monthly Syphilis and 6-monthly HIV testing. We estimated the correlates of regular STI and HIV testing using negative binomial regression.

**Results:**

Of the respondents 420 (95.2%) reported to have ever taken an HIV test with 297 (67.4%) testing two or more times in the prior 12 months. Over half of the respondents (59%) reported ever taking a syphilis test with only 62 (14.1%) reporting testing three or more times in the prior 12 months. After adjusting for socio-demographics, attitude and norms, high perceived self-efficacy was associated with a 33% increase in the likelihood of repeated HIV testing [prevalence ratio (PR), 1.33, 95% confidence interval (CI) 1.15–1.53] while low perceived confidence was associated with a 25% decrease in the likelihood of repeated HIV testing (PR, 0.75, 95% CI 0.63–0.89). Similarly low attitudes and norms were associated with a decrease of 52.6% (PR, 0.47, 95% CI 0.37–0.61) and 47% (PR, 0.53, 95% CI 0.41–0.69) in the likelihood of repeated syphilis testing respectively.

**Conclusion:**

Compared to HIV, uptake of repeated syphilis testing was very low. Correlates of HIV testing include; perceived self-efficacy amidst barriers and perceived confidence for HIV and low attitudes and accepting norms for syphilis. Health campaigns should emphasize overcoming barriers to HIV testing while promoting attitudes and norms including integration of serological syphilis testing and other STIs into HIV services.

## Background

The burden of HIV and other sexually transmitted infections (STIs) is disproportionately high among female sex workers globally [1, 2]. Female sex workers (FSW) are 13.5 times (95% CI 10.0–18.1) as likely to be living with HIV as compared to women of reproductive age in the general population [2]. HIV prevalence is substantially higher among FSW in sub-Saharan Africa (SSA) (36.9%) than globally (11.8–30.7%) [2, 3]. Eastern and southern Africa is the region most affected by the HIV epidemic [4, 5], accounting for 45% of new HIV infections globally [5]. In 2017, approximately 2–8% of new HIV infections in this region occurred among sex workers and their clients [5]. Importantly, FSW are a bridge population—up to 15% of HIV infections in the general female adult population are attributable to sex work [6]. In Uganda, sex work is estimated to contribute 7–11% of new HIV infections [7]. HIV prevalence among FSW 32.4–52% [8–10] is significantly higher than truck drivers (25%), fisher folk (22%), uniformed personnel (18%) and women in the general population (7.3%) [11–13].

The disproportionate HIV burden in African sex workers is engendered by STI co-infection. Evidence shows that in 2014, STI prevalence ranged from 24.9% to 77.5%; between half and two-thirds of FSW have a curable STI at any one time, 30% have reactive syphilis serology and 10% or more have an active genital ulcer [14]. A recent study in Tanzania also found high burden of syphilis (8%) and herpes simplex (HSV) type 2 (60%) [15]. Similar findings have been reported in Ugandan studies that have found high burden of STIs including herpes simplex virus (HSV) type 2 (80%), gonorrhea (13%) and chlamydia (9%) [8, 10]. Several studies have shown that concurrent STI infections increase HIV acquisition and transmission risk. This increased risk has been demonstrated for both ulcerative and non-ulcerative STIs [16–19]. In a prospective study of 242 high-risk women in South Africa, presence of a concurrent STI was associated with a threefold increased risk of HIV infection [hazard ratio (HR) 3.29; 95% CI 1.5–7.2] [20]. This increased risk is mediated through a variety of biological mechanisms including inflammatory cytokines, changes in vaginal flora, recruitment of local CD4 positive cells, and disruption of genital and rectal mucosal barriers [8, 21–25].

The World Health Organization (WHO) recommends STI screening for FSW every 3–6 months and HIV testing every 6–12 months [26]. Regular screening for STIs and HIV services is an important entry point for treatment and prevention programs [27, 28], but individual, structural, societal and policy barriers limit uptake [29]. There is low uptake of STI and HIV screening services among FSW in Uganda; only 53% of FSW had previously tested for HIV in one study [8]. Limited data are available regarding regularity and correlates of STI and HIV screening among FSW in SSA [8, 15, 30].

In this study, we aimed to assess the frequency of STI and HIV screening and the psychosocial correlates associated with screening among FSW in Uganda. Psychosocial factors including behavioural intentions, attitudes, social norms, perceived efficacy, perceived severity and normative beliefs among others have received recognition as predictors of health behaviour [31–34]. Derived from common psychosocial theories like theory of planned [32, 34, 35], behavioural intentions refers to an individual’s readiness to perform a given behaviour or plans to perform a given behaviour in the future, for example testing for syphilis every 3 months or every 6 months for HIV. Attitudes consists an individual’s overall evaluation of a health behaviour that is beliefs about the consequences of repeated syphilis and HIV testing, social norms refers to a person’s own estimate of the social pressure to perform or not perform the target behaviour, while self-efficacy refers to the extent to which a person feels able to enact or perform a given behaviour [32].

## Methods

### Study design and setting

Between July and October 2018, we conducted a cross-sectional survey of 441 FSW in the municipalities of Arua, Mbale, Mbarara and Kampala (combined general population 1,857,088) [36] representing the four major regions (Northern, Eastern, Western and Central) of Uganda to describe STI and HIV testing practices. Study participants included women ≥ 17 years engaged in sex work according to self-report of selling sex for goods or money for at least 6 months. We used a two-stage sampling design to recruit study participants.

### Respondents’ recruitment procedures

Prior to participant recruitment, a mapping exercise was conducted to gain an understanding of time, hot spots, typologies, sex work procurer connections and territorial management in each municipality. From the mapping exercise, the main typologies based on venue were: street, lodge, and bar/club, and brothel (mainly in Kampala). Sex work hotspots constituted the primary sampling units (PSUs). Based on the different typologies, lists were drawn to form a sampling frame for each municipality. In Kampala, we randomly selected 20 PSUs, consisting of brothels, lodges, streets and bar/club. In Mbarara, Arua and Mbale, we randomly selected between 10 and 15 PSUs consisting mainly of lodges, street and bar/club. Between 7 and 10 participants were recruited from each PSU in each municipality.

Sampling began with 2–3 FSW at each PSUs, identified through key informants (bar maids, pimps and managers). The recruits were given a brief training on peer recruitment and 2–3 paper coupons. Each coupon contained an identification number, contact information of the research team and the duration of the survey in the municipality. Only respondents who presented the coupon after verification and met the eligibility criteria were consented to participate in an interviewer-administered questionnaire. The questionnaire is included in the supplemental methods (see Additional file 1). At the end of the interview the recruits received coupons and information for peer recruitment.

All study respondents received information about STI and HIV screening. Ethical clearance for the study was obtained Makerere from University School of Public Health Research and Ethics Committee and Uganda National Council for Science and Technology (Ref: HS 2403). All respondents provided written consent in their local language.

### Statistical analysis

The primary outcome was count of STI and HIV testing in the prior 12 months, defined as number of serological tests for HIV, syphilis and other STIs in the prior 12 months. The major independent variables were categorized as attitude or motivators, self-efficacy, moral norms or normative and social influences. These categories were derived from constructs of the Integrated Change Model [32, 37]. Attitude in this study referred to beliefs about the individualized merits and demerits of testing for every 3 months for syphilis and 6 months for HIV. Self-efficacy referred to individualized perceived level of confidence and control about testing for HIV every 6 months amidst barriers (stigma, discrimination, fear of positive results, privacy and confidentiality) and 3 monthly syphilis testing. Social norms referred to approval or social expectation of 3-monthly syphilis testing and 6-monthly HIV testing by significant others (peers and clients). Moral norms were conceptualized as whether a FSW thought it was her obligation and a necessity for her to seek 3 monthly syphilis testing and 6 monthly HIV testing and whether her fellow FSW are seeking regular testing. Each category was assessed using question items on a 6-point Likert scale. The distribution of testing frequency was skewed and overdispersed with variance greater than 1. The overdispersion was due to excess number of zero testing frequencies in the prior 12 months, compared to normal Poisson distribution. Negative binomial regression, a model recommended for overdispersed count data, was used to fit the data [38]. We used principal component analysis (PCA) to reduce 29 items used in the questionnaire to measure major independent variables.

The Kaiser–Meyer–Olkin measure verified the sampling adequacy for the analysis, KMO of 0.90, and all KMO values for individual items were greater than 0.85, well above the limit of 0.5 (field, 2009). Bartlett’s test of sphericity X2 (435) of 5669.642, p < 0.001, indicated the correlations between the items were large enough for PCA. Nine factors had eigenvalues over Kaiser’s criterion of 1 and all together explained 66.175% of the variance. The items that clustered on the same components suggested that the factors represented Intention to test for HIV in the next 6 months (IN-HIV), Intention to test for syphilis in the next 3 months (IN-SY), attitude towards 6 monthly HIV testing (AT-HIV), Attitude towards 3 monthly syphilis testing (AT-SY), social approval of 6 monthly HIV testing (SN), Moral norm towards testing every 3 months for syphilis (MN_SY), Moral norm towards testing for HIV every 6 months (MN-HIV), Perceived efficacy to go for 3 monthly syphilis checkups (SE-SY) and Perceived efficacy to go for 6 monthly HIV testing (SE_HIV). Using Cronbach’s α statistic, we assessed the reliability of item scales and computed scale medians (Table 3). Participants were considered to score high on the item scale that is attitude, if their score is above the scale median.

For this paper we describe how attitude, accepting norms and self-efficacy influences the frequency of syphilis and HIV testing and our next publication will describe intentions. We evaluated the correlates of self-reported frequency of STI and HIV testing (count data) using negative binomial regression (for over dispersed count data). We used a likelihood ratio test of alpha = 0 (dispersion parameter) to assess model fit and found no evidence of over-dispersion. Crude and adjusted prevalence ratios (aPR) and 95% confidence intervals (CI) were estimated. Statistical analyses were performed using SPSS version 23.0 (IBM Corp, Armonk, NY) and Stata version 12.0 (StataCorp, College Station, TX).

## Results

### Socio-demographic characteristics

The median age of the participants was 26 years [interquartile range (IQR), 23–30] (Table 1). Just over half (52.8%) of the participants had obtained primary education and 10.2% had no formal education at all. The median duration in sex work was 32 months (IQR, 17–60), with lodge (36%), street (32.8%), bar/club (23.5%) and brothels (3.4%) being the common venue of operation. A high proportion (42.4%) described themselves as separated, 82.1% were biological mothers, and over half (58.7%) reported consistent condom use during work.

**Table 1:** Socio-demographic characteristics of participants (N = 441)

Variable	Frequency (%)	Median (IQR)
Age (years)		26 (23–30)
Duration in sex work (months)		32 (17–60)
Biological children		2 (1–3)
Other dependants		1 (0–2)
Education level		
None	45 (10.2)	
Primary	233 (52.8)	
Secondary	153 (34.7)	
Higher education	10 (2.3)	
Marital status		
Married	26 (5.9)	
Separated	187 (42.4)	
Widow	26 (5.9)	
Never married	100 (22.7)	
Have a boyfriend	102 (23.1)	
Typology by venue		
Street	306 (32.8)	
Home	36 (3.9)	
Lodge	336 (36.0)	
Bar/club	220 (23.5)	
Brothel	32 (3.4)	
Escort	4 (0.4)	
Mobility		
Work only this town	330 (74.8)	
Move regularly in many towns in Uganda	99 (22.4)	
Move regularly in other towns outside Uganda	12 (2.7)	
Condom use		
All the time with all clients	259 (58.7)	
Inconsistent use	182 (41.3)	

### STI and HIV testing behaviors of respondents

A significant number (47.2%, n = 208) of respondents reported past medical history of syphilis (Table 2). The life time testing for HIV among respondents was high at 95.2% (n = 420), with 67.4% (n = 297) testing two or more times in the last 12 months. The lifetime testing for other STIs like syphilis was 59% (n = 260) and only 14.1% (n = 62) reporting testing three or more times in the last 12 months. The commonly cited reason for not testing for syphilis included: having no signs or pains (34%), having never thought about it (21%), self-medication in case of signs (20.6%), not being aware (11%), consistent condom use (6.7%), not aware of where to obtain the service (4%) and costs (2.2%).

**Table 2:** STI and HIV testing behaviors among participants (N = 441)

Variable	Arua n = 101	Kampala n = 140	Mbale n = 100	Mbarara n = 100	Total (%)
Ever tested for syphilis/STI					
Yes	49	100	61	50	260 (59)
No	51	40	39	50	181 (41)
Testing frequency in the last 12 months					
Never	58	53	55	61	227 (51.5)
Once	25	35	23	14	97 (22.0)
Twice	8	20	17	10	55 (12.5)
Three times	8	26	3	12	49 (11.0)
Four or more	2	6	2	3	13 (3.0)
Ever tested for HIV					
Yes	97	135	93	95	420 (95)
No	4	5	7	5	21 (5.0)
Testing frequency in the last 12 months					
Never	6	21	20	13	60 (13.6)
Once	19	27	19	19	84 (19.0)
Twice	17	23	23	29	92 (20.9)
Three times	31	49	24	26	130 (29.)
Four or more	28	20	14	13	75 (17.0)
History of syphilis					
I don’t know	39	7	4	5	55 (12.4)
No	48	52	39	39	178 (40.4)
Yes	14	81	57	56	208 (47.2)
History of gonorrhoea					
I don’t know	23	8	1	5	37 (8.4)
No	75	98	68	77	318 (72.1)
Yes	3	34	31	18	86 (19.5)
History of genital herpes					
I don’t know	33	21	11	7	72 (16.3)
No	62	104	82	88	336 (76.2)
Yes	6	15	7	5	33 (7.5)

### Respondents’ scores on the scale dimensions derived from PCA

Among respondents, there was low attitude scores towards syphilis testing compared to HIV. The median score for 3-monthly syphilis testing was 9 (IQR, 6–10) compared to 21 (IQR, 21–22) for HIV (Table 3). Similarly respondents’ scores (median = 9, IQR, 7–11) on 3-monthly syphilis testing as a moral norm were low compared to 6-monthly HIV testing scores (median = 17, IQR, 15–19). Over half of FSW (59.6%, n = 263) scored low on perceived self-efficacy amidst barriers (fear of positive result, stigma and discrimination and lack of privacy and confidentiality) to seek 6-monthly HIV testing. Only 37.2% (n = 164) agree or strongly agree to being able to seek 3-monthly syphilis testing from a health facility.

**Table 3:** Participants scores on attitudes, norms and self-efficacy towards regular STI and HIV testing

Variable	Median (IQR)	Freq < median (%)	Freq > median (%)
AT_HIV, attitude to 6 monthly testing	21 (20–22)	298 (67.6)	143 (32.4)
AT-SY, attitude to 3 monthly testing	9 (6–10)	269 (61.0)	172 (39.0)
MN-SY, syphilis testing accepting norms	9 (7–11)	269 (61.0)	172 (39.0)
MN_HIV, HIV testing accepting norms	17 (15–19)	255 (57.8)	186 (42.2)
SEB_HIV, efficacy to test for HIV amidst barriers	12 (9–14)	263 (59.6)	178 (40.4)
PC_HIV, confidence to test for HIV every 6 months	20 (18–21)	142 (32.2)	299 (67.8)

*AT-HIV* attitude to 6-monthly HIV testing, *AT-SYP* attitude to 3-monthly syphilis/STI testing, *MN* normative or descriptive norm, *SEB* perceived self-efficacy amidst barriers, *PC* perceived confidence

### Negative binomial multivariable model for syphilis testing frequency in the last 12 months

After adjusting for socio-demographics (age, level of education, and marital status), condom use practices, as well as self-efficacy, the correlates of repeated syphilis testing were: attitude, moral norm and town location (see Table 4). When comparing FSW based on their attitude scores, the likelihood of repeated syphilis testing was 52.6% lower among FSW whose attitude scores were lower than the attitude scale median (aPR = 0.47; 95% CI 0.37–0.61). Similarly, there was a 47% decrease in the likelihood of repeated testing among FSW with low normative scores (aPR = 0.53; 95% CI 0.41–0.69) compared to FSW with normative scores above the scale median. FSW in Arua had a 33% decreased (aPR = 0.67; 95% CI 0.49–0.92) chances as compared to Kampala of having regular syphilis testing.

**Table 4:** Negative binomial multivariable model for syphilis testing frequency in the last 12 months

	Crude ratios			Adjusted ratios		
	PR	95% CI	p-value	PR	95% CI	p-value
Age category in years						
17–19	Reference					
20–23	1.756	0.98–3.15	0.059	1.594	0.861–2.949	0.138
24–28	2.278	1.29–3.99	0.004	1.827	1.000–3.335	0.050
29–33	2.164	1.19–3.93	0.011	1.786	0.921–3.466	0.086
34–38	2.337	1.26–4.34	0.007	1.781	0.895–3.547	0.100
39 and above	2.509	1.073–5.87	0.034	1.679	0.637–4.421	0.294
Level of education						
None	Reference					
Primary	1.153	0.797–1.667	0.449	1.055	0.700–1.590	0.796
Secondary	1.515	1.043–2.199	0.029	1.163	0.763–1.772	0.482
Higher education	0.955	0.422–2.158	0.911	0.913	0.375–2.219	0.840
Marital status						
Married	Reference					
Separated	0.708	0.485–1.035	0.074	0.961	0.625–1.479	0.858
Widow	0.812	0.484–1.363	0.432	1.129	0.629–2.027	0.683
Single	0.772	0.517–1.152	0.205	0.997	0.621–1.603	0.992
Have a regular boy friend	0.725	0.485–1.084	0.117	0.895	0.563–1.421512	0.638
Town location						
Kampala	Reference					
Mbarara	0.645	0.496–0.838	0.001	1.008	0.726–1.398	0.962
Mbale	0.582	0.444–0.763	0.001	0.926	0.649–1.320	0.672
Arua	0.568	0.433–0.746	0.001	0.672	0.488–0.924	0.015*
Condom use description						
Consistently use with all clients	Reference					
Doesn’t use sometimes	0.831	0.679–1.016	0.071	0.990	0.790–1.240	0.931
ATT_SYP						
Score > median (9)	Reference					
Score < median (9)	0.343	0.279–0.420	0.001	0.474	0.369–0.608	0.001*
MN_SYP						
Score > median (9)	Reference					
Score < median (9)	0.362	0.295–0.443	0.001	0.528	0.406–0.688	0.001*
PC_SYP						
Strongly disagree	Reference					
Disagree	1.763	0.907–3.427	0.094	1.459	0.721–2.952	0.293
Somewhat disagree	1.684	0.873–3.250	0.120	1.367	0.6801–2.747	0.380
Somewhat agree	1.835	0.950–3.545	0.071	1.435	0.709–2.903	0.315
Agree	1.898	0.998–3.610	0.051	1.206	0.603–2.409	0.596
Strongly agree	3.111	1.561–6.20	0.001	1.860	0.877–3.945	0.106

### Negative binomial multivariable model for HIV testing frequency in the last 12 months

After adjusting for socio-demographics (age, level of education, and marital status), condom use practices, as well as attitude, moral norms, and social norms, the correlates of repeated HIV testing were: perceived self-efficacy amidst barriers (SEB_HIV), confidence and town location (see Table 5). The likelihood of repeated HIV testing was 33% higher (aPR = 1.33, 95% CI 1.15–1.53) among FSW who scored high on perceived self-efficacy amidst barriers compared to those who scored low, while the likelihood of repeated testing was 25% lower among FSW who scored low on perceived confidence (aPR = 0.75, 95% CI 0.63–0.89) compared to those who scored high. Also FSW residing in Arua were more likely to have regular HIV testing when compared to the ones residing in Kampala (aPR = 1.280, 95% CI 1.061–1.545).

**Table 5:** negative binomial multivariable model for HIV testing frequency in the last 12 months

	Crude ratios			Adjusted ratios		
	PR	95% CI	p-value	PR	95% CI	p-value
Age in completed years						
17–19	Reference					
20–23	1.102	0.825–1.472	0.509	1.111	0.828–1.492	0.481
24–28	1.148	0.871–1.512	0.328	1.131	0.848–1.509	0.403
29–33	1.093	0.805–1.486	0.568	1.127	0.805–1.577	0.486
34–38	0.981	0.699–1.377	0.913	1.029	0.714–1.484	0.877
39 and above	1.272	0.764–2.117	0.355	1.410	0.820–2.426	0.214
Level of education						
None	Reference					
Primary	1.063	0.848–1.332	0.594	0.948	0.749–1.200286	0.659
Secondary	1.183	0.938–1.492	0.156	1.048	0.8208–1.337	0.709
Higher education	1.062	0.659–1.708	0.805	0.918	0.5616–1.500	0.733
Marital status						
Married	Reference					
Separated	0.973	0.743–1.275	0.844	0.974	0.736–1.288	0.851
Widow	0.717	0.484–1.060	0.095	0.724	0.483–1.085	0.117
Single	0.962	0.723–1.279	0.790	0.929	0.687–1.257	0.635
Have a regular boy friend	0.904	0.679–1.205	0.494	0.909	0.673–1.229	0.539
Town location						
Kampala	Reference					
Mbarara	0.966	0.809–1.153	0.702	1.151	0.946–1.399	0.159
Mbale	0.901	0.752–1.079	0.257	1.048	0.858–1.279	0.648
Arua	1.192	1.001–1.407	0.039	1.280	1.061–1.545	0.010*
Condom use description						
Consistently use with all clients	Reference					
Doesn’t use sometimes	0.936	0.822–1.065	0.313	0.945	0.826–1.081	0.412
ATT_HIV						
Score > median (21)	Reference					
Score < median (21)	0.790	0.693–0.900	0.001	0.883	0.763–1.021	0.094
MN_HIV						
Score > median (17)	Reference					
Score < median (17)	0.809	0.713–0.919	0.001	0.927	0.809–1.063	0.277
PSEB_HIV						
Score < median (12)	Reference					
Score > median (12)	1.453	1.280–1.649	0.001	1.328	1.148–1.535	0.001*
PC_HIV						
Score > median (20)	Reference					
Score < median (20)	0.624	0.537–0.726	0.001	0.749	0.629–0.889	0.001*
Peer approval (FSW)						
Neither approve nor disapprove	Reference					
Disapprove strongly	1.133	0.874–1.469	0.346	1.120	0.856–1.467	0.408
Disapprove somewhat	1.148	0.916–1.439	0.231	1.076	0.851–1.362	0.539
Approve somewhat	1.292	1.046–1.595	0.018	1.153	0.919–1.448	0.219
Approve	1.314	1.059–1.631	0.013	1.151	0.910–1.455	0.240
Approve strongly	1.435	0.969–2.126	0.072	1.291	0.862–1.933	0.216

## Discussion

This study focused on the regularity of Syphilis and HIV screening practices and their associated correlates among Female Sex Workers (FSW). The findings of the study show that 95.2% of the FSW reported ever testing for HIV with 67.4% testing two or more times in the last 12 months preceding the survey. Relatedly, 59% reported ever testing for syphilis with only 14.1% testing three or more times in the last 12 months. The Health Organization (WHO) recommends 3–6 monthly screening for STIs like syphilis and 6–12 monthly HIV testing strategy [28, 39–42] as a key entry point into treatment, care and prevention [27, 28]. Despite availability of HIV and STIs testing, treatment and management for FSW in Uganda [29], the current findings indicate very low uptake of regular syphilis testing (1 in 10) compared to 7 in 10 for HIV. Our finding that 67.4% of FSW report testing two or more times was high compared to those previously reported in a number of studies in SSA in the same population [8, 30, 43]. Previous studies in Kampala reported that HIV testing in the prior 12 months was 16% among FSW [8] while another study in Benin found a 47% prevalence of HIV testing in 12 months among FSW [33].

Not much is documented on testing rates for syphilis. However, the high testing rates for HIV compared to syphilis could partly be explained by attitudes, accepting norms perceived self-efficacy and regular HIV outreach services for FSW. We observed that compared to HIV, FSW had low scores on attitudes, accepting norms and self-efficacy towards 3-monthly syphilis testing. Among the FSW, high scores on perceived self-efficacy amidst barriers (fear, stigma and discrimination, confidentiality) was associated with an increase of 33% in the likelihood of regular HIV testing while low confidence was associated with a decreased of 25%. The study findings are consistent with research evidence that fear of positive results, perceptions of stigma and discrimination, perceived lack of privacy and confidentiality are barriers to regular uptake of HIV testing among FSW [29, 30] but also that attitudes, norms and self-efficacy influence STI and HIV testing behavior [30]. In contrast, low attitudes and norms were associated with a decreased of 56.2% and 47% in likelihood of regular syphilis testing respectively. Attitudes consists of perceived cognitive and emotional merits and demerits of a particular health behavior, including beliefs that the health behavior can contribute to a lowered risk of developing a disease [32, 34, 37]. Thus the findings could suggests low understanding of the benefits of regular syphilis testing among FSW.

Reasons cited for not screening for syphilis included; having no signs and symptoms, having never thought about it, not aware that you can screen, presence of self-medication in case of signs and symptoms, consistent condom use, not knowing where to obtain the service and costs. Some of the reasons cited above point to limited knowledge about syphilis and screening practices. Furthermore, in Uganda programming tends to focus on HIV both at policy and service delivery with regard to serological screening. For example, while not specific to FSW there exists a national policy guidelines on HIV testing services [44], but syndromic management guidelines for other STIs [45]. These policy practices could influence norms of both providers and service users.

The findings of this study support the theorization that psychosocial factors: attitude, norms and perceived self-efficacy are determinants of health behavior. Also significant in this study was town location, compared to FSW in Kampala, residing in Arua was associated with a decrease of 33% in the likelihood of syphilis screening but an increase of 28% in the likelihood of HIV screening. Location is described in literature to influence access to and utilization of services [30, 46]. The differences in service organization (service providers, timing and costs), socio-economic and demographic characteristics could partly explain the differences in screening rates. Kampala is a capacity city, and compared to Arua has many organizations including private clinics that offer STI screening services. But also Kampala has for long been the focus for HIV and STI prevention campaigns including moonlight outreaches for FSW by many non-governmental organizations. However, of late a number of organizations are now expanding their outreaches to upcountry municipalities like Arua. Thus the observation that more FSW were likely to be screened for syphilis in Kampala could be explained by availability of wider range of providers including private clinics. However, the observation that FSW were more likely to test for HIV could be attributed to availability of outreach services in Arua and fatigue in Kampala.

There are some study limitations related to the design and representativeness. The participants were recruited from the municipalities of Kampala, Mbarara, Mbale and Arua and as such may not be representative of all the municipalities and communities where FSW work in Uganda. It was observed during data collection that these sites have a regular active moonlight HIV Counseling and Testing outreach program. The study design was cross-sectional and findings could differ over a period of time. In addition, social desirability bias in reporting HIV testing behavior, the lifetime HIV testing prevalence of 95% among FSW was high compared to less than 60% reported in a recent Kampala study. However, our findings support the model that low attitudes and norms decrease the chances of regular syphilis screening while high perceived self-efficacy amidst barriers increases the chances of regular HIV testing among FSW. Overcoming barriers and promotion of attitudes and norms should be key in STI and HIV promotional campaigns and programs. At clinical level there is need to emphasize the integration of STI testing into HIV services.

## Conclusion

Compared to HIV, the uptake of repeated syphilis screening was low among town-based FSW in Uganda, and was significantly positively associated with low attitudes, norms and town location. The psychosocial correlates of repeated HIV testing were perceived self-efficacy and confidence. Health campaigns should emphasize overcoming barriers to HIV testing while promoting attitudes and norms including integration of serological syphilis testing and other STIs into HIV services.

## Supplementary information

**Additional file 1.** Questionnaire for female sex workers; Quantitative survey among sex workers in Uganda on STI and HIV screening intentions, practices and psychosocial predictors.

## Data Availability

The dataset used during the current study are available from the corresponding author on request. However, the questionnaire is included in this published article as a digital supplementary information.

## Abbreviations

FSW: female sex workers
HIV: human immunodeficiency virus
IQR: interquartile range
PCA: principal component analysis
PSU: primary sampling unit
STI: sexually transmitted infection
WHO: World Health Organization
