# Supplementary material for: Psychosocial correlates of regular syphilis and HIV screening practices among female sex workers in Uganda: a cross-sectional survey
Source: AIDS Res Ther. 2019 Sep 18;16:28. doi: 10.1186/s12981-019-0244-0 (PMC6751878; doi:10.1186/s12981-019-0244-0)
Supplement: Supplementary file 1 — Additional file 1. Questionnaire for female sex workers; Quantitative survey among sex workers in Uganda on STI and HIV screening intentions, practices and psychosocial predictors. [file 12981_2019_244_MOESM1_ESM.docx]

**Questionnaire for female sex workers; Quantitative survey among sex workers in Uganda on STI and HIV screening intentions, practices & psychosocial predictors**

SECTION: SOCIODEMOGRAPHIC CHARACTERISTICS

| No. | Questions and filters | Answers and codes | Shift to |
| --- | --- | --- | --- |
|  | Sex | Male……………………….1  Female……………………..2 |  |
| Q01 | Age in complete years |  |  |
| Q02 | Highest level of education attained; primary, secondary, higher education  *Interviewer, circle only one answer* | No education…………..0  Primary …………………….1  Secondary……………………2  Higher education……………..3 |  |
| Q03 | Marital status  *Interviewer, list all the possibilities. There can be more than one answer circled* | Married…………………..1  Separated………………….2  Widow…………………….3  Single………………………4  Have a boyfriend…………...5 |  |
| Q04 | Number (biological) of children currently |  |  |
| Q05 | Number of people other than biological children presently under your care |  |  |
| Q06 | Duration in sex work in completed months |  |  |
| Q07 | How would describe your current work  *Interviewer, list all the possibilities. There can be more than one answer circled* | Full time, as I have no other sources of income……………………….1  Part-time, as I have other sources of income……………………….2  Part-time, as I am a student……..3  Full-time, to supplement my other sources of income  Others specify………………………. |  |
| Q08 | Venue of operation  *Interviewer, list all the possibilities. There can be more than one answer circled* | Street ……………………………………..1  Home………………………………………2  Lodge……………………………………….3  Bar…………………………………………..4  Brothel………………………………………5  Others specific………………………………. |  |
|  | Description of work by client type (for male sex workers only | My clients are only female………………..1  My clients are only male………………….2  My clients are both male and female………3 |  |
| Q09 | Mobility during work  *Interviewer, list all the possibilities. There can be more than one answer circled* | Specific site in this town only………………1  Only in this town but move in many sites…..2  Move regularly in many towns in Uganda…..3  Move regularly to other towns outside Uganda……………………………………..4  Others specify……………………………. |  |
| Q09 | Condom use at last sexual intercourse | Yes……………………….1  No………………………….2 | 2 9 |
| Q9 | Reasons for not using a condom at last sexual intercourse.  *Interviewer, many answers are possible. List all as mentioned by the participant* |  |  |
| Q10 | How would you describe your condom use practice in your work  *Interviewer, many answers are possible* | I consistently use condom at every sexual intercourse with all my clients…………………………….1  I sometimes don’t use a condom with my regular client/s…………2  I sometimes don’t use a condom if the client requests so……………3  Sometimes if the client offers more for condom less sex………………4  I sometimes don’t use a condom if I like the client by look …………5  Others specify…………………….. |  |

| Q011 | Past history of common STIs.  *Interviewer, tell the participant the she will be provided a small anonymous card to provide this information and that she can fill the card in privacy and put in an envelope.* |  |  |
| --- | --- | --- | --- |

**SECTION; STI AND HIV RISK PERCEPTION**

| No. | Questions and filters | Answers and codes | Shift to |
| --- | --- | --- | --- |
| Q012 | In your view, how serious is contracting an STIs (syphilis, gonorrhea, and herpes) as a health problem  *Interviewer, list all the choices and circle one answer* | Not very serious…………..1  Not serious…………………2  Slightly serious…………….3  Neither serious nor very serious……………………..4  Serious …………………….5  Very serious………………..6 |  |
| Q13 | **Current level of risk** of being infected with an STI like syphilis  *Interviewer, list all the choices and circle one answer* | Very low……………….1  Low…………………….2  Slightly low…………….3  Neither low nor high……4  High……………………..5  Very high………………...6 |  |
| Q14 | How serious is contracting HIV as a health problem | Not very serious…………..1  Not serious…………………2  Slightly serious…………….3  Neither serious nor very serious……………………..4  Serious …………………….5  Very serious……………….. |  |
| Q15 | Current level of risk of being infected with HIV if not already infected | Very low……………….1  Low…………………….2  Slightly low…………….3  Neither low nor high……4  High……………………..5  Very high………………...6 |  |
| Q16 | Reasons you evaluate your current level of risk that way  *Interviewer, many possible answers.* | Many sexual partners……….1  Consistent condoms use…….2  Inconsistent condom use……3  Others specify……………… |  |
| Q17 | In your view, what is your future risk of contracting HIV if you don’t regularly test for STIs and HIV | Very low……………….1  Low…………………….2  Slightly low…………….3  High……………………..4  Very high………………...5  I don’t know------------------6 |  |
| Q18 | Having an STI increases your risk of contracting HIV | Strongly disagree---------------1  Disagree------------------------2  Neither disagree nor agree----3  Agree----------------------------4  Strongly agree------------------5  I don’t know--------------------6 |  |

**SECTION; STI AND HIV TESTING BEHAVIOR**

| Q19 | I don’t want to know the result but have you already gone to test for STIs other than HIV (*interviewer; give examples of STIs*)  If yes when was the last screening done | Yes ……………………………1  No………………………………2  During the last 3 months………..1  During the last 6 months…………2  During the last 12 months………..3  Between 12 months and 3 years ago………………………………...4  More than 3 years ago…………….5 | **2 Q22** |
| --- | --- | --- | --- |
| Q20 | Where (public health facility, private clinic, outreach) did you have the last screening done | Place; **________________________** |  |
| Q21 | During the last 12 months, how often did you screen for STIs | Never……………………………….1  Once………………………………..2  Twice………………………………3  Three times………………………...4  Four times or more…………………5 |  |
| Q22 | Reasons you went to screen for STIs.  *Interviewer, many possible answers* | To know my status………………….1  I do it as a routine practice………….2  I was not feeling well……………….3  On request or recommendation of health worker……………………….4  Had signs of an STI…………………5  Others specify ………………………. |  |
| Q23 | Why have you never gone to screen for STIs  Interviewer, list all reasons mentioned |  |  |
| Q24 | Don’t tell me the result, but have you already gone for an HIV test  If yes when was the testing done | Yes--------------------------------1  No---------------------------------2  During the last 3 months-------------1  During the last 6 months-------------2  During the last 12 months-----------3  Between 12 months and 3 years ago-------------------------------------------4  More than 3 years ago…………….5 | **2 q28** |
| Q25 | Where (public health facility, private clinic, outreach) did you have the last screening done | Place; **________________________** |  |
| Q26 | During the last 12 months, how often did you test for HIV | Never---------------------------------1  Once----------------------------------2  Twice---------------------------------3  Three times--------------------------4  Four times or more------------------5 |  |
| Q27 | Reasons you went to test for HIV.  *Interviewer, list all mentioned* |  |  |
| Q28 | Don’t tell me the results, but did you obtain the results of your HIV test  If no, why?  Interviewer, list all reasons given | Yes------------------------------------1  No-------------------------------------2 |  |
| Q29 | Why have you never gone to test for HIV  **Interviewer, circle all mentioned** | Fear of the result------------------------1  Fear of the reaction of others-----------2  Don’t need one because I am not sick --------------------------------------------------3  I don’t have time--------------------------4  I don’t seek the use-----------------------5  Others (specify) ................................... |  |
| Q30 | Preferences of where you would want to have an STI and HIV tests done. | Places;______________________ |  |

**SECTION; Major independent variables**

Introduction: This section is reserved for HIV negative people or those unware of their HIV or STI status.

The questions focus on the intention to go for STI screening in the next 3 to 6 months and HIV testing in the next 6 to 12 months.

**Interviewer**; advice participants to response to the best of their knowledge. Specify to them that there are no right or wrong answer. The most important thing is to say how the situation described apply to you. So please answer the question by saying what you really think.

Interviewer; Explain and demonstrate scale to the participant so that she can see and chose a response on the scale for each item.

**Section: intention**

| No. | Statement | Answers and codes |
| --- | --- | --- |
| To what extend do you agree or disagree with the following statements | | |
| Q31 | You intend to go for Syphilis screening during the next 3-6 months | Strongly disagree---------------1  Disagree------------------------2  Somewhat disagree------------3  Somewhat agree----------------4  Agree-----------------------------5  Strongly agree------------------6 |
| Q32 | You are going to be screened for syphilis during the next 3-6 months | Strongly disagree---------------1  Disagree------------------------2  Somewhat disagree------------3  Somewhat agree----------------4  Agree-----------------------------5  Strongly agree------------------6 |
| Q33 | How would you evaluate your chances that you would be screened for Syphilis in the next 3-6 months | Neither low nor high--------------1  Very low----------------------------2  Low----------------------------------3  Moderate----------------------------4  High---------------------------------5  Very high………………………6 |
| Q34 | You intend to go for HIV testing during the next 6-12 months | Strongly disagree---------------1  Disagree------------------------2  Somewhat disagree------------3  Somewhat agree----------------4  Agree-----------------------------5  Strongly agree------------------6 |
| Q35 | You are going to be tested for HIV during the next 6-12 months | Strongly disagree---------------1  Disagree------------------------2  Somewhat disagree------------3  Somewhat agree----------------4  Agree-----------------------------5  Strongly agree------------------6 |
| Q36 | How would you evaluate your chances that you would be tested for HIV in the next 6-12 months | Neither low nor high--------------1  Very low----------------------------2  Low----------------------------------3  Moderate----------------------------4  High---------------------------------5  Very high………………………6 |
| 37 | If you do not intent to go an Syphilis checkup in the next 3-6 months, kindly what are your reasons? *Interviewer; list all mentioned* |  |
| 38 | If you do not intent to go an HIV test in the next 6-12 months, kindly what are your reasons?  *Interviewer; list all mentioned* |  |

**Section; Attitudes and behavioral beliefs**

| No. | Statement | Answers and codes |
| --- | --- | --- |
| Q39 | For you to get screened for Syphilis every 3-6 months would be; | Neither harmful nor beneficial------------------1  Very harmful---------------2  Harmful----------------------3  Slightly beneficial…………4  Beneficial----------------------5  Very beneficial----------------6 |
| Q40 | For you to get tested for HIV every 6-12 months would be; | Neither harmful nor beneficial------------------1  Very harmful---------------2  Harmful----------------------3  Slightly beneficial…………4  Beneficial----------------------5  Very beneficial----------------6 |
| Q41 | For you, getting screened for Syphilis every 3-6 months, would reduce your risk of contracting HIV | Strongly disagree-------------1  Disagree-----------------------2  Somewhat disagree-----------3  Somewhat agree--------------4  Agree---------------------------5  Strongly agree-----------------6 |
| Q42 | For you, getting tested for HIV every 6-12 months, would allow you to be better informed about your health | Strongly disagree-------------1  Disagree-----------------------2  Somewhat disagree-----------3  Somewhat agree--------------4  Agree---------------------------5  Strongly agree-----------------6 |
| Q43 | For, you getting tested for HIV every 6-12 months would help you better protect your self | Strongly disagree-------------1  Disagree----------------------2  Somewhat disagree-----------3  Somewhat agree--------------4  Agree---------------------------5  Strongly agree-----------------6 |
| Q44 | You would fill proud if you are tested for HIV every 6-12 months | Strongly disagree-------------1  Disagree-----------------------2  Somewhat disagree-----------3  Somewhat agree--------------4  Agree---------------------------5  Strongly agree-----------------6 |
| Q45 | If you get tested for HIV every 6-12 months, it would allow you to access treatment and care for HIV in case of a positive result | Strongly disagree-------------1  Disagree-----------------------2  Somewhat disagree-----------3  Somewhat agree--------------4  Agree---------------------------5  Strongly agree-----------------6 |

**Section; Normative beliefs**

| No. | Statement | Answers and codes |
| --- | --- | --- |
| In your opinion, to what extent would the people or groups who are important to you approve or disapprove if you announced to them you would be going for HIV testing every 6-12 months | | |
| **Q**46 | Friends/fellow sex workers | Neither approval nor disapprove--------------------1  Disapprove strongly----------2  Disapprove somewhat--------3  Approve somewhat-----------4  Approve……………………..5  Strongly approve---------------6 |
| **Q47** | Regular partners/boy friends | Neither approval nor disapprove--------------------1  Disapprove strongly----------2  Disapprove somewhat--------3  Approve somewhat-----------4  Approve……………………..5  Strongly approve---------------6 |

**Section: Moral and descriptive norms**

| No | Statement | Answers and codes |
| --- | --- | --- |
| To what extend to you agree with the following statements | | |
| Q48 | When you are a sex worker, it is necessary to go for Syphilis screening every 3-6 months | Strongly disagree-------------1  Disagree----------------------2  Somewhat disagree-----------3  Somewhat agree--------------4  Agree---------------------------5  Strongly agree-----------------6 |
| Q49 | When you are a sex worker, it is necessary to go for HIV testing every 6-12 months | Strongly disagree-------------1  Disagree----------------------2  Somewhat disagree-----------3  Somewhat agree--------------4  Agree---------------------------5  Strongly agree-----------------6 |
| Q50 | Being tested for HIV every 6-12 month is a moral obligation for you as a sex worker | Strongly disagree-------------1  Disagree----------------------2  Somewhat disagree-----------3  Somewhat agree--------------4  Agree---------------------------5  Strongly agree-----------------6 |
| Q51 | Being screened for Syphilis every 3-6 months is a normal routine that many sex workers practice | Strongly disagree-------------1  Disagree----------------------2  Somewhat disagree-----------3  Somewhat agree--------------4  Agree---------------------------5  Strongly agree-----------------6 |
| Q52 | Being tested for HIV every 6-12 months is a normal routine that many sex workers practice | Strongly disagree-------------1  Disagree----------------------2  Somewhat disagree-----------3  Somewhat agree--------------4  Agree---------------------------5  Strongly agree-----------------6 |
| Q53 | Based on what your known about your fellow SWs and the practice of STI screening, how many of them are being screened every 3-6 months for Syphilis | None------------------------1  Minority (25%)--------------2  Half (50%)--------------------3  Majority (75%)----------------4  All (100%)---------------------5 |
| Q54 | Based on what your known about your fellow SWs and the practice of HIV testing, how many of them are being screened every 6-12 months for HIV | None------------------------1  Minority (25%)--------------2  Half (50%)--------------------3  Majority (75%)----------------4  All (100%)---------------------5 |

**Section: Self-efficacy and control beliefs**

| No. | Statement | Answers and codes |
| --- | --- | --- |
| Q54 | I am confident I can go for STI screening every 3 to 6 months | Strongly disagree-------------1  Disagree----------------------2  Somewhat disagree-----------3  Somewhat agree--------------4  Agree---------------------------5  Strongly agree-----------------6 |
| Q55 | For you to be tested for HIV every 6-12 months would be; | Neither difficult nor easy----1  Very difficult------------------2  Somewhat difficult-----------3  Somewhat easy----------------4  Easy----------------------------5  Very easy----------------------6 |
| Q56 | I am confident I can go for HIV testing every 6-12 months | Strongly disagree-------------1  Disagree----------------------2  Somewhat disagree-----------3  Somewhat agree--------------4  Agree---------------------------5  Strongly agree-----------------6 |
| Q57 | Do you feel able to go for an HIV test every 6-12 months, even if you are afraid of receiving a positive result | Strongly disagree-------------1  Disagree----------------------2  Somewhat disagree-----------3  Somewhat agree--------------4  Agree---------------------------5  Strongly agree-----------------6 |
| Q58 | Do you feel able to go for an HIV test every 6-12 months, despite fear of discrimination and stigmatization in case of a positive result | Strongly disagree-------------1  Disagree----------------------2  Somewhat disagree-----------3  Somewhat agree--------------4  Agree---------------------------5  Strongly agree-----------------6 |
| Q59 | Do you feel able to go for an HIV test every 6-12 months, even if you don’t know completely if this information will remain confidential | Strongly disagree-------------1  Disagree----------------------2  Somewhat disagree-----------3  Somewhat agree--------------4  Agree---------------------------5  Strongly agree-----------------6 |
| Q60 | If HIV testing is free, you will go for HIV testing every 6-12 months | Strongly disagree-------------1  Disagree----------------------2  Somewhat disagree-----------3  Somewhat agree--------------4  Agree---------------------------5  Strongly agree-----------------6 |
| Q61 | You would go for HIV testing every 6-12 months, if you know where the service is offered | Strongly disagree-------------1  Disagree----------------------2  Somewhat disagree-----------3  Somewhat agree--------------4  Agree---------------------------5  Strongly agree-----------------6 |
| Q62 | You would go for HIV testing every 6-12 months, if you got a reminder message | Strongly disagree-------------1  Disagree----------------------2  Somewhat disagree-----------3  Somewhat agree--------------4  Agree---------------------------5  Strongly agree-----------------6 |
| Q63 | I would be willing to receive on my phone an SMS message reminder to go for regular STI and HIV screening | Strongly disagree-------------1  Disagree----------------------2  Somewhat disagree-----------3  Somewhat agree--------------4  Agree---------------------------5  Strongly agree-----------------6 |
| Q64 | If I receive an SMS reminder message from my preferred clinic, I would go for STI and HIV screening | Strongly disagree-------------1  Disagree----------------------2  Somewhat disagree-----------3  Somewhat agree--------------4  Agree---------------------------5  Strongly agree-----------------6 |
| Q65 | I would prefer a reminder message from a fellow sex worker to go for STI and HIV screening than a mobile phone message | Strongly disagree-------------1  Disagree----------------------2  Somewhat disagree-----------3  Somewhat agree--------------4  Agree---------------------------5  Strongly agree-----------------6 |

This is the end of our questionnaire. Thank you very much for your time in answering these questions.

We appreciate your assistance.
